# Supplementary material for: Recent Evidences of Epigenetic Alterations in Chronic Obstructive Pulmonary Disease (COPD): A Systematic Review
Source: Int J Mol Sci. 2025 Mar 13;26(6):2571. doi: 10.3390/ijms26062571 (PMC11942187; doi:10.3390/ijms26062571)
Supplement: Supplementary file 1 [file ijms-26-02571-s001.zip › ijms-3489783-supplementary.pdf]

PRISMA 2020 Checklist

Supplementary Table S1: PRISMA checklist; [12]

Section and Topic	Ite m #	Checklist item	Location where item is reported
**TITLE**			
Title	1	Identify the report as a systematic review.	1
**ABSTRACT**			
Abstract	2	See the PRISMA 2020 for Abstracts checklist.	2
**INTRODUCTION**			
Rationale	3	Describe the rationale for the review in the context of existing knowledge.	3
Objectives	4	Provide an explicit statement of the objective(s) or question(s) the review addresses.	3
**METHODS**			
Eligibility criteria	5	Specify the inclusion and exclusion criteria for the review and how studies were grouped for the syntheses.	13-14
Information sources	6	Specify all databases, registers, websites, organisations, reference lists and other sources searched or consulted to identify studies. Specify the date when each source was last searched or consulted.	13-14
Search strategy	7	Present the full search strategies for all databases, registers and websites, including any filters and limits used.	Tables 7-9
Selection process	8	Specify the methods used to decide whether a study met the inclusion criteria of the review, including how many reviewers screened each record and each report retrieved, whether they worked independently, and if applicable, details of automation tools used in the process.	13-14
Data collection process	9	Specify the methods used to collect data from reports, including how many reviewers collected data from each report, whether they worked independently, any processes for obtaining or confirming data from study investigators, and if applicable, details of automation tools used in the process.	13-14
Data items	10a	List and define all outcomes for which data were sought. Specify whether all results that were compatible with each outcome domain in each study were sought (e.g. for all measures, time points, analyses), and if not, the methods used to decide which results to collect.	13-14
	10b	List and define all other variables for which data were sought (e.g. participant and intervention characteristics, funding sources). Describe any assumptions made about any missing or unclear information.	/
Study risk of bias assessment	11	Specify the methods used to assess risk of bias in the included studies, including details of the tool(s) used, how many reviewers assessed each study and whether they worked independently, and if applicable, details of automation tools used in the process.	Supplementar y Table 2
Effect measures	12	Specify for each outcome the effect measure(s) (e.g. risk ratio, mean difference) used in the synthesis or presentation of results.	/
Synthesis methods	13a	Describe the processes used to decide which studies were eligible for each synthesis (e.g. tabulating the study intervention characteristics and comparing against the planned groups for each synthesis (item #5)).	13-14
	13b	Describe any methods required to prepare the data for presentation or synthesis, such as handling of missing summary statistics, or data conversions.	13-14
	13c	Describe any methods used to tabulate or visually display results of individual studies and syntheses.	13-14
	13d	Describe any methods used to synthesize results and provide a rationale for the choice(s). If meta-analysis was performed, describe the model(s), method(s) to identify the presence and extent of statistical heterogeneity, and software package(s) used.	/
	13e	Describe any methods used to explore possible causes of heterogeneity among study results (e.g. subgroup analysis, meta-regression).	13-14
	13f	Describe any sensitivity analyses conducted to assess robustness of the synthesized results.	/
Reporting bias assessment	14	Describe any methods used to assess risk of bias due to missing results in a synthesis (arising from reporting biases).	13-14
Certainty	15	Describe any methods used to assess certainty (or confidence) in the body of evidence for an outcome.	/

assessment			
**RESULTS**			
Study selection	16a	Describe the results of the search and selection process, from the number of records identified in the search to the number of studies included in the review, ideally using a flow diagram.	4
	16b	Cite studies that might appear to meet the inclusion criteria, but which were excluded, and explain why they were excluded.	4
Study characteristics	17	Cite each included study and present its characteristics.	4-11
Risk of bias in studies	18	Present assessments of risk of bias for each included study.	/
Results of individual studies	19	For all outcomes, present, for each study: (a) summary statistics for each group (where appropriate) and (b) an effect estimate and its precision (e.g. confidence/credible interval), ideally using structured tables or plots.	Tables 1-6
Results of syntheses	20a	For each synthesis, briefly summarise the characteristics and risk of bias among contributing studies.	Supplementary table 2
	20b	Present results of all statistical syntheses conducted. If meta-analysis was done, present for each the summary estimate and its precision (e.g. confidence/credible interval) and measures of statistical heterogeneity. If comparing groups, describe the direction of the effect.	4-11; Tables 1-6
	20c	Present results of all investigations of possible causes of heterogeneity among study results.	/
	20d	Present results of all sensitivity analyses conducted to assess the robustness of the synthesized results.	/
Reporting biases	21	Present assessments of risk of bias due to missing results (arising from reporting biases) for each synthesis assessed.	/
Certainty of evidence	22	Present assessments of certainty (or confidence) in the body of evidence for each outcome assessed.	/
**DISCUSSION**			
Discussion	23a	Provide a general interpretation of the results in the context of other evidence.	12
	23b	Discuss any limitations of the evidence included in the review.	12
	23c	Discuss any limitations of the review processes used.	12
	23d	Discuss implications of the results for practice, policy, and future research.	12
**OTHER INFORMATION**			
Registration and protocol	24a	Provide registration information for the review, including register name and registration number, or state that the review was not registered.	13-14
	24b	Indicate where the review protocol can be accessed, or state that a protocol was not prepared.	13-14
	24c	Describe and explain any amendments to information provided at registration or in the protocol.	/
Support	25	Describe sources of financial or non-financial support for the review, and the role of the funders or sponsors in the review.	14-15
Competing interests	26	Declare any competing interests of review authors.	14-15
Availability of data, code and other materials	27	Report which of the following are publicly available and where they can be found: template data collection forms; data extracted from included studies; data used for all analyses; analytic code; any other materials used in the review.	14

Supplementary Table S2: AXIS tool								
**The Appraisal tool for Cross-Sectional Studies (AXIS tool, 2016) Part 1**								
Study	Burke H, et al 2022	Cazola-Rivero S, et al 2020	Chang C, et al 2024	Chen Q, et al 2022	Cordero AIH, et al, 2022	Dai Z, et al, 2022	De Smet E, et al 2020	Ding Y, et al 2023
Introduction								
1. Were the aims/objectives of the study clear?	YES	YES	YES	YES	YES	YES	YES	YES
Methods								
2. Was the study design appropriate for the stated aim(s)?	YES	YES	YES	YES	YES	YES	YES	YES
3. Was the sample size justified?	YES	YES	NO	YES	YES	NO	YES	YES
4. Was the target/reference population clearly defined? (Is it clear who the research was about?)	YES	YES	YES	YES	YES	YES	YES	YES
5. Was the sample frame taken from an appropriate population base so that it closely represented the target/reference population under investigation?	YES	YES	YES	YES	YES	YES	YES	YES
6. Was the selection process likely to	YES	YES	YES	YES	YES	YES	YES	YES

select subjects/participants that were representative of the target/reference population under investigation?								
7. Were measures undertaken to address and categorise non-responders?	NO	NO	NO	NO	NO	NO	NO	NO
8. Were the risk factor and outcome variables measured appropriate to the aims of the study?	YES	YES	YES	YES	YES	YES	YES	YES
9. Were the risk factor and outcome variables measured correctly using instruments/ measurements that had been trialled, piloted or published previously?	YES	YES	YES	YES	YES	YES	YES	YES
10. Is it clear what was used to determined statistical significance and/or	YES	YES	YES	YES	YES	YES	YES	YES

precision estimates? (eg, p values, CIs)								
11. Were the methods (including statistical methods) sufficiently described to enable them to be repeated?	YES	YES	YES	YES	YES	YES	YES	YES
Results								
12. Were the basic data adequately described?	YES	YES	YES	YES	YES	YES	YES	YES
13. Does the response rate raise concerns about non-response bias?	NO	NO	NO	NO	NO	NO	NO	NO
14. If appropriate, was information about non-responders described?	NON-APPROPRIATE	NON-APPROPRIATE	NON-APPROPRIATE	NON-APPROPRIATE	NON-APPROPRIATE	NON-APPROPRIATE	NON-APPROPRIATE	NON-APPROPRIATE
15. Were the results internally consistent?	YES	YES	YES	YES	YES	YES	YES	YES
16. Were the results for the analyses described in the methods, presented?	YES	YES	YES	YES	YES	YES	YES	YES

The Appraisal tool for Cross-Sectional Studies (AXIS tool, 2016) Part 2								
Study	Duan R, et al, 2020	Feng X, et al 2023	Günes GG, et al, 2022	Hu J, et al 2022	Huang H, et al 2020	Kachroo P, et al 2020	Kachroo P, et al 2021	Kim R, et al 2021
Introduction								
1. Were the aims/objectives of the study clear?	YES	YES	YES	YES	YES	YES	YES	YES
Methods								
2. Was the study design appropriate for the stated aim(s)?	YES	YES	YES	YES	YES	YES	YES	YES
3. Was the sample size justified?	YES	NO	YES	NO	YES	YES	YES	NO
4. Was the target/reference population clearly defined? (Is it clear	YES	YES	YES	YES	YES	YES	YES	YES

who the research was about?)								
5. Was the sample frame taken from an appropriate population base so that it closely represented the target/reference population under investigation?	YES	YES	YES	YES	YES	YES	YES	YES
6. Was the selection process likely to select subjects/participants that were representative of the target/reference population under investigation?	YES	YES	YES	YES	YES	YES	YES	YES
7. Were measures undertaken to address and categorise non-responders?	NO	NO	NO	NO	NO	NO	NO	NO
8. Were the risk factor and outcome variables measured appropriate to the aims of the study?	YES	YES	YES	YES	YES	YES	YES	YES

9. Were the risk factor and outcome variables measured correctly using instruments/ measurements that had been trialled, piloted or published previously?	YES	YES	YES	YES	YES	YES	YES	YES
10. Is it clear what was used to determined statistical significance and/or precision estimates? (eg, p values, CIs)	YES	YES	YES	YES	YES	YES	YES	YES
11. Were the methods (including statistical methods) sufficiently described to enable them to be repeated?	YES	YES	YES	YES	YES	YES	YES	YES
Results								
12. Were the basic data adequately described?	YES	YES	YES	YES	YES	YES	YES	YES
13. Does the response rate raise	NO	NO	NO	NO	NO	NO	NO	NO

concerns about non-response bias?								
14. If appropriate, was information about non-responders described?	NON-APPROPRIATE	NON-APPROPRIATE	NON-APPROPRIATE	NON-APPROPRIATE	NON-APPROPRIATE	NON-APPROPRIATE	NON-APPROPRIATE	NON-APPROPRIATE
15. Were the results internally consistent?	YES	YES	YES	YES	YES	YES	YES	YES
16. Were the results for the analyses described in the methods, presented?	YES	YES	YES	YES	YES	YES	YES	YES

**The Appraisal tool for Cross-Sectional Studies (AXIS tool, 2016) Part 3**								
Study	Li B, et al 2023	Liu P, et al 2022	Liu P, et al, 2021	Liu S, et al, 2020	Morrow JD, et al 2020	Nadi E, et al 2022	Schwartz U, et al 2023	Shen H, et al 2021
Introduction								

1. Were the aims/objectives of the study clear?	YES	YES	YES	YES	YES	YES	YES	YES
Methods								
2. Was the study design appropriate for the stated aim(s)?	YES	YES	YES	YES	YES	YES	YES	YES
3. Was the sample size justified?	NO	NO	YES	YES	YES	YES	NO	YES
4. Was the target/reference population clearly defined? (Is it clear who the research was about?)	YES	YES	YES	YES	YES	YES	YES	YES
5. Was the sample frame taken from an appropriate population base so that it closely represented the target/reference population under investigation?	YES	YES	YES	YES	YES	YES	YES	YES
6. Was the selection process likely to select subjects/participan ts that were representative of the	YES	YES	YES	YES	YES	YES	YES	YES

target/reference population under investigation?								
7. Were measures undertaken to address and categorise non-responders?	NO	NO	NO	NO	NO	NO	NO	NO
8. Were the risk factor and outcome variables measured appropriate to the aims of the study?	YES	YES	YES	YES	YES	YES	YES	YES
9. Were the risk factor and outcome variables measured correctly using instruments/ measurements that had been trialled, piloted or published previously?	YES	YES	YES	YES	YES	YES	YES	YES
10. Is it clear what was used to determined statistical significance and/or precision estimates? (eg, p values, CIs)	YES	YES	YES	YES	YES	YES	YES	YES

11. Were the methods (including statistical methods) sufficiently described to enable them to be repeated?	YES	YES	YES	YES	YES	YES	YES	YES
Results								
12. Were the basic data adequately described?	YES	YES	YES	YES	YES	YES	YES	YES
13. Does the response rate raise concerns about non-response bias?	NO	NO	NO	NO	NO	NO	NO	NO
14. If appropriate, was information about non-responders described?	NON-APPROPRIATE	NON-APPROPRIATE	NON-APPROPRIATE	NON-APPROPRIATE	NON-APPROPRIATE	NON-APPROPRIATE	NON-APPROPRIATE	NON-APPROPRIATE
15. Were the results internally consistent?	YES	YES	YES	YES	YES	YES	YES	YES
16. Were the results for the analyses described in the methods, presented?	YES	YES	YES	YES	YES	YES	YES	YES

The Appraisal tool for Cross-Sectional Studies (AXIS tool, 2016) Part 4								
Study	Shen X, et al, 2024	Shen Y, et al 2021	Shi X, et al 2022	Singh P, et al 2024	Strom JE, et al 2022	Tang S, et al,2023	Tao S, et al 2024	Tasena H, et al 2022
Introduction								
1. Were the aims/objectives of the study clear?	YES	YES	YES	YES	YES	YES	YES	YES
Methods								
2. Was the study design appropriate for the stated aim(s)?	YES	YES	YES	YES	YES	YES	YES	YES
3. Was the sample size justified?	YES	YES	YES	NO	NO	YES	YES	NO
4. Was the target/reference population clearly defined? (Is it clear who the research was about?)	YES	YES	YES	YES	YES	YES	YES	YES
5. Was the sample frame taken from an appropriate population base so that it closely represented the target/reference population under investigation?	YES	YES	YES	YES	YES	YES	YES	YES
6. Was the selection process likely to select	YES	YES	YES	YES	YES	YES	YES	YES

subjects/participants that were representative of the target/reference population under investigation?								
7. Were measures undertaken to address and categorise non-responders?	NO	NO	NO	NO	NO	NO	NO	NO
8. Were the risk factor and outcome variables measured appropriate to the aims of the study?	YES	YES	YES	YES	YES	YES	YES	YES
9. Were the risk factor and outcome variables measured correctly using instruments/ measurements that had been trialled, piloted or published previously?	YES	YES	YES	YES	YES	YES	YES	YES
10. Is it clear what was used to determined statistical significance and/or	YES	YES	YES	YES	YES	YES	YES	YES

precision estimates? (eg, p values, CIs)								
11. Were the methods (including statistical methods) sufficiently described to enable them to be repeated?	YES	YES	YES	YES	YES	YES	YES	YES
Results								
12. Were the basic data adequately described?	YES	YES	YES	YES	YES	YES	YES	YES
13. Does the response rate raise concerns about non-response bias?	NO	NO	NO	NO	NO	NO	NO	NO
14. If appropriate, was information about non-responders described?	NON-APPROPRIATE	NON-APPROPRIATE	NON-APPROPRIATE	NON-APPROPRIATE	NON-APPROPRIATE	NON-APPROPRIATE	NON-APPROPRIATE	NON-APPROPRIATE
15. Were the results internally consistent?	YES	YES	YES	YES	YES	YES	YES	YES
16. Were the results for the analyses described in the methods, presented?	YES	YES	YES	YES	YES	YES	YES	YES

The Appraisal tool for Cross-Sectional Studies (AXIS tool, 2016) Part 5								
Study	Wang C, et al 2021	Wang F, et al 2023	Wang L, et al 2022	Wang Y, et al,2020	Wang Z, et al,2021	Wu S, et al, 2020	Xie T, et al, 2024	Yang H, et al 2021
Introduction								
1. Were the aims/objectives of the study clear?	YES	YES	YES	YES	YES	YES	YES	YES
Methods								
2. Was the study design appropriate for the stated aim(s)?	YES	YES	YES	YES	YES	YES	YES	YES
3. Was the sample size justified?	YES	YES	NO	YES	YES	YES	NO	YES
4. Was the target/reference population clearly defined? (Is it clear who the research was about?)	YES	YES	YES	YES	YES	YES	YES	YES
5. Was the sample frame taken from an appropriate population base so that it closely represented the target/reference population under investigation?	YES	YES	YES	YES	YES	YES	YES	YES

6. Was the selection process likely to select subjects/participants that were representative of the target/reference population under investigation?	YES	YES	YES	YES	YES	YES	YES	YES
7. Were measures undertaken to address and categorise non-responders?	NO	NO	NO	NO	NO	NO	NO	NO
8. Were the risk factor and outcome variables measured appropriate to the aims of the study?	YES	YES	YES	YES	YES	YES	YES	YES
9. Were the risk factor and outcome variables measured correctly using instruments/ measurements that had been trialled, piloted or published previously?	YES	YES	YES	YES	YES	YES	YES	YES
10. Is it clear what was used to	YES	YES	YES	YES	YES	YES	YES	YES

determined statistical significance and/or precision estimates? (eg, p values, CIs)								
11. Were the methods (including statistical methods) sufficiently described to enable them to be repeated?	YES	YES	YES	YES	YES	YES	YES	YES
Results								
12. Were the basic data adequately described?	YES	YES	YES	YES	YES	YES	YES	YES
13. Does the response rate raise concerns about non-response bias?	NO	NO	NO	NO	NO	NO	NO	NO
14. If appropriate, was information about non-responders described?	NON-APPROPRIATE	NON-APPROPRIATE	NON-APPROPRIATE	NON-APPROPRIATE	NON-APPROPRIATE	NON-APPROPRIATE	NON-APPROPRIATE	NON-APPROPRIATE
15. Were the results internally consistent?	YES	YES	YES	YES	YES	YES	YES	YES
16. Were the results for the analyses described	YES	YES	YES	YES	YES	YES	YES	YES

in the methods, presented?								

The Appraisal tool for Cross-Sectional Studies (AXIS tool, 2016) Part 6								
Study	Yang Z, et al 2023	Zhang C, et al, 2022	Zhang J, et al 2020	Zhang L, et al 2022	Zhang X, et al 2022	Zhang Z, et al 2021	Zhao S, et al,2021	Zheng L, et al 2021
Introduction								
1. Were the aims/objectives of the study clear?	YES	YES	YES	YES	YES	YES	YES	YES
Methods								
2. Was the study design appropriate for the stated aim(s)?	YES	YES	YES	YES	YES	YES	YES	YES
3. Was the sample size justified?	NO	YES	YES	NO	YES	NO	YES	YES
4. Was the target/reference population clearly defined? (Is it clear who the research was about?)	YES	YES	YES	YES	YES	YES	YES	YES
5. Was the sample frame taken from an appropriate population base so that it closely represented the	YES	YES	YES	YES	YES	YES	YES	YES

target/reference population under investigation?								
6. Was the selection process likely to select subjects/participants that were representative of the target/reference population under investigation?	YES	YES	YES	YES	YES	YES	YES	YES
7. Were measures undertaken to address and categorise non-responders?	NO	NO	NO	NO	NO	NO	NO	NO
8. Were the risk factor and outcome variables measured appropriate to the aims of the study?	YES	YES	YES	YES	YES	YES	YES	YES
9. Were the risk factor and outcome variables measured correctly using instruments/ measurements that had been trialled, piloted or	YES	YES	YES	YES	YES	YES	YES	YES

published previously?								
10. Is it clear what was used to determined statistical significance and/or precision estimates? (eg, p values, CIs)	YES	YES	YES	YES	YES	YES	YES	YES
11. Were the methods (including statistical methods) sufficiently described to enable them to be repeated?	YES	YES	YES	YES	YES	YES	YES	YES
Results								
12. Were the basic data adequately described?	YES	YES	YES	YES	YES	YES	YES	YES
13. Does the response rate raise concerns about non-response bias?	NO	NO	NO	NO	NO	NO	NO	NO
14. If appropriate, was information about non-responders described?	NON-APPROPRIATE	NON-APPROPRIATE	NON-APPROPRIATE	NON-APPROPRIATE	NON-APPROPRIATE	NON-APPROPRIATE	NON-APPROPRIATE	NON-APPROPRIATE

15. Were the results internally consistent?	YES	YES	YES	YES	YES	YES	YES	YES
16. Were the results for the analyses described in the methods, presented?	YES	YES	YES	YES	YES	YES	YES	YES

**The Appraisal tool for Cross-Sectional Studies (AXIS tool, 2016) Part 7**			
Study	Zhou Ai, et al, 2020	Zhu Y, et al 2022	Zong D, et al, 2022
Introduction			
1. Were the aims/objectives of the study clear?	YES	YES	YES
Methods			
2. Was the study design appropriate for the stated aim(s)?	YES	YES	YES
3. Was the sample size justified?	NO	NO	NO
4. Was the target/reference population clearly defined? (Is it clear who the research was about?)	YES	YES	YES

5. Was the sample frame taken from an appropriate population base so that it closely represented the target/reference population under investigation?	YES	YES	YES
6. Was the selection process likely to select subjects/participants that were representative of the target/reference population under investigation?	YES	YES	YES
7. Were measures undertaken to address and categorise non-responders?	NO	NO	NO
8. Were the risk factor and outcome variables measured appropriate to the aims of the study?	YES	YES	YES
9. Were the risk factor and outcome variables measured correctly using	YES	YES	YES

instruments/ measurements that had been trialled, piloted or published previously?			
10. Is it clear what was used to determined statistical significance and/or precision estimates? (eg, p values, CIs)	YES	YES	YES
11. Were the methods (including statistical methods) sufficiently described to enable them to be repeated?	YES	YES	YES
Results			
12. Were the basic data adequately described?	YES	YES	YES
13. Does the response rate raise concerns about non-response bias?	NO	NO	NO
14. If appropriate, was information about non-responders described?	NON-APPROPRIATE	NON-APPROPRIATE	NON-APPROPRIATE

15. Were the results internally consistent?	YES	YES	YES
16. Were the results for the analyses described in the methods, presented?	YES	YES	YES

PRISMA 2020 for Abstracts Checklist

Supplementary Table S3: PRISMA abstract checklist [12]

Section and Topic	Item #	Checklist item	Reported (Yes/No)
**TITLE**			
Title	1	Identify the report as a systematic review.	yes
**BACKGROUND**			
Objectives	2	Provide an explicit statement of the main objective(s) or question(s) the systematic review addresses.	yes
**METHODS**			
Eligibility criteria	3	Specify the inclusion and exclusion criteria for the systematic review.	no
Information sources	4	Specify the information sources (e.g. databases, registers) used to identify studies and the date when each was last searched.	yes
Risk of bias	5	Specify the methods used to assess risk of bias in the included studies.	no
Synthesis of results	6	Specify the methods used to present and synthesise results.	yes
**RESULTS**			
Included studies	7	Give the total number of included studies and participants and summarise relevant characteristics of studies.	yes
Synthesis of results	8	Present results for main outcomes, preferably indicating the number of included studies and participants for each. If systematic review was done, report the summary estimate and confidence/credible interval. If comparing groups, indicate the direction of the effect (i.e. which group is favoured).	yes
**DISCUSSION**			
Limitations of evidence	9	Provide a brief summary of the limitations of the evidence included in the systematic review (e.g. study risk of bias, inconsistency and imprecision).	yes
Interpretation	10	Provide a general interpretation of the results and important implications.	yes
**OTHER**			
Funding	11	Specify the primary source of funding for the systematic review.	no
Registration	12	Provide the register name and registration number.	no
